# P-367. Containment of an Outbreak of Carbapenem-Resistant *Acinetobacter baumanii* in a Burn Intensive Care Unit

**DOI:** 10.1093/ofid/ofae631.568

**Published:** 2025-01-29

**Authors:** Saman Kannangara, Richard F Grossman, Cheryl Sharp, Marice lejano-Shao, Pureum Lee, Isabel Jiang, Erwin R Gotico

**Affiliations:** Saint Francis Memorial Hospital/UCSF Health, San Francisco, California; Saint Francis Hospital/Dignity Health, San Francisco, California; Saint Francis Memorial Hospital, Fort Worth, Texas; CSUEB, San Francisco, California; Saint Francis Memorial Hospital, Fort Worth, Texas; Saint Francis Memorial Hospital, Fort Worth, Texas; CommonSpirit, San Francisco, California

## Abstract

**Background:**

*Acinetobacter baumanii* is a cause of serious infections in hospital settings. Carbapenem-resistant *Acinetobacter baumanii* (CRAB) is especially difficult to treat. Patients in a burn unit are particularly vulnerable to colonization and infection with few reports describing CRAB outbreaks in a burn intensive care unit and measures required for containment. This study highlights the infection control measures successful in controlling an outbreak.

**Methods:**

After detection of a patient with CRAB in an urban burn unit in January 2023, all patients in the burn unit were screened for carbapenem resistant organisms. Six patients with CRAB were identified between January 2023 and May 2023. Whole genomic sequencing was used for confirmation of clonal relatedness. All patients with a positive CRAB culture of sterile or non-sterile sites are included in this retrospective study.

**Results:**

Six patients ages 29-53 tested positive for CRAB between January 2023 and May 2023. Case 1 was admitted from a long term acute care facility, with sputum culture positive for CRAB. One had CRAB from a blood culture, and four in wound cultures. Longer length of stay, 80 days vs 13 days was seen with CRAB positive patients because of partial skin graft failure, slower to heal wounds, and barriers to placement. After judicious use of antibiotics, following infection control practices were implemented:

1. Screening for carbapenem resistant organisms (CPOs) on admission, weekly, and on discharge.

2. Fluorescent markers added to adenosine triphosphate (ATP) testing of environmental surfaces and equipment.

3. Double terminal cleaning of rooms with CRAB.

4. A cleaning process for every piece of equipment.

5. Hand hygiene education and proper use of personal protective equipment.

With the above measures, no cases of CRAB were seen after May 2023.

**Conclusion:**

The CRAB outbreak was associated with patients with burn wounds, antibiotic use, similarities in age and demographics, slower wound healing, partial skin graft loss, and longer length of stay. The control measures listed were successful in controlling the outbreak.

**Disclosures:**

**All Authors**: No reported disclosures

